# Magnetic Resonance and Computed Tomography Findings of Isolated Hydatid Cyst of the Interventricular Septum

**DOI:** 10.5334/jbsr.1670

**Published:** 2018-11-23

**Authors:** Deniz Alis, Onder Turna

**Affiliations:** 1 Istanbul Mehmet Akif Ersoy Thoracic and Cardiovascular Surgery Training and Research Hospital, TR

**Keywords:** Cyst hydatid, Cardiac MRI, Cardiac CT, Cardiac cyst hydatid

## Case

**Figure 1:**
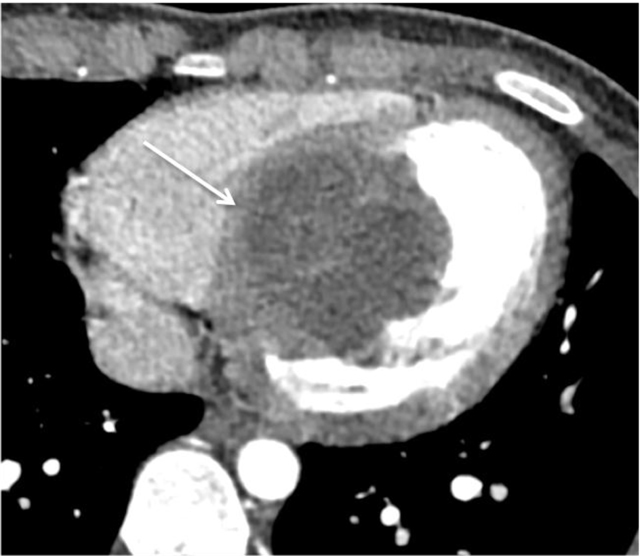
The contrast-enhanced CT image of the patient shows hypodense mass lesion with a size of 5.5 × 6 × 5.5 cm in the interventricular septum (arrow). The lesion has thick and slightly irregular capsule with internal septations.

**Figure 2:**
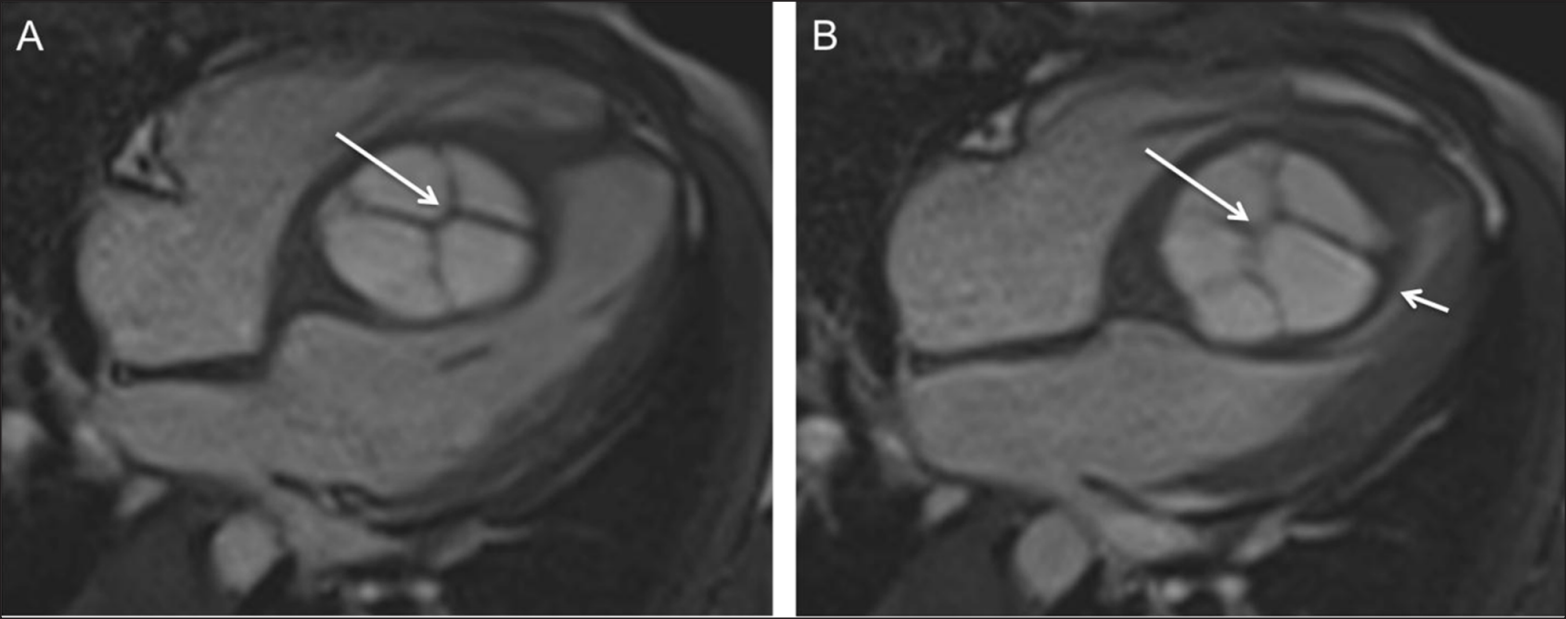
The four chamber view images of the patient on steady-state free precision MRI during the diastole **(a)** and the systole **(b)**. The lesion has hyperintense signal with internal septations. Also, note that the mid-ventricular and apical part of the left ventricular cavity is substantially obliterated by the mass during the systole (short arrow).

An 18-year-old male patient was admitted to our cardiology department with shortness of breath and chest pain on exertion lasting for one year. His blood profile, biochemical analyses, and physical examination were normal. Echocardiography revealed cystic mass with internal septations in the interventricular septum protruding into the left ventricular cavity. Prospective electrocardiography (ECG) gated cardiac computed tomography (CT) and magnetic resonance imaging (MRI) was performed for further characterization of the mass. Contrast-enhanced CT of the patient demonstrated a hypodense mass with a size of 5.5 × 6 × 5.5 cm in the interventricular septum (arrow) (Figure 1). The four chamber view images of the patient on steady-state free precision MRI during the diastole (Figure 2a) and the systole (Figure 2b) depicted that the lesion mostly involved the mid-ventricular and the apical parts of the interventricular septum and had hyperintense signal characteristics with internal septations (arrows). Also, the lesion substantially obliterated the left ventricular cavity during the systole (short arrow) without causing significant obstruction at the ventricular outflow tract (Figure 2b). On provisional diagnosis of hydatid cyst, blood serology was requested, which yielded positive results for Echinococcus granulosus. Further imaging studies, which were ordered for the possible other locations that might be involved by hydatid cysts, yielded negative results. The patient refused any medical or surgical treatment and was discharged of his own will.

## Comment

Isolated cardiac cyst hydatid is an uncommon disease with an estimated prevalence ranging from 0.02 to 2% [1]. Most cases are asymptomatic, yet it might manifest with angina, pulmonary hypertension, arrhythmia, and anaphylactic reactions. The cysts preferentially involve the left ventricle given to rich vascularization. Echocardiography is commonly the initial modality for the diagnosis. Cardiac CT and MRI might serve to entail a detailed characterization of the cysts and to assess the relationship of the mass with the surrounding cardiac or extracardiac tissues [1]. Surgical excision of the cyst with medical treatment is the most common treatment strategy for the cardiac hydatid cysts, which yielded favorable outcomes in the previous cases [1].
